# Prostate Specific Membrane Antigen (PSMA) Regulates Angiogenesis Independently of VEGF during Ocular Neovascularization

**DOI:** 10.1371/journal.pone.0041285

**Published:** 2012-07-18

**Authors:** Christina L. Grant, Leslie A. Caromile, Khayyam Durrani, M. Mamunur Rahman, Kevin P. Claffey, Guo-Hua Fong, Linda H. Shapiro

**Affiliations:** Center for Vascular Biology, University of Connecticut Health Center, Farmington, Connecticut, United States of America; University of Florida, United States of America

## Abstract

**Background:**

Aberrant growth of blood vessels in the eye forms the basis of many incapacitating diseases and currently the majority of patients respond to anti-angiogenic therapies based on blocking the principal angiogenic growth factor, vascular endothelial growth factor (VEGF). While highly successful, new therapeutic targets are critical for the increasing number of individuals susceptible to retina-related pathologies in our increasingly aging population. Prostate specific membrane antigen (PSMA) is a cell surface peptidase that is absent on normal tissue vasculature but is highly expressed on the neovasculature of most solid tumors, where we have previously shown to regulate angiogenic endothelial cell invasion. Because pathologic angiogenic responses are often triggered by distinct signals, we sought to determine if PSMA also contributes to the pathologic angiogenesis provoked by hypoxia of the retina, which underlies many debilitating retinopathies.

**Methodology/Principal Findings:**

Using a mouse model of oxygen-induced retinopathy, we found that while developmental angiogenesis is normal in PSMA null mice, hypoxic challenge resulted in decreased retinal vascular pathology when compared to wild type mice as assessed by avascular area and numbers of vascular tufts/glomeruli. The vessels formed in the PSMA null mice were more organized and highly perfused, suggesting a more ‘normal’ phenotype. Importantly, the decrease in angiogenesis was not due to an impaired hypoxic response as levels of pro-angiogenic factors are comparable; indicating that PSMA regulation of angiogenesis is independent of VEGF. Furthermore, both systemic and intravitreal administration of a PSMA inhibitor in wild type mice undergoing OIR mimicked the PSMA null phenotype resulting in improved retinal vasculature.

**Conclusions/Significance:**

Our data indicate that PSMA plays a VEGF-independent role in retinal angiogenesis and that the lack of or inhibition of PSMA may represent a novel therapeutic strategy for treatment of angiogenesis-based ocular diseases.

## Introduction

It is estimated that by 2050 nearly 15% of the global population will be 65 years of age or older [1]. When coupled with the escalating prevalence of diabetes worldwide, this increase in lifespan has prompted predictions of a striking rise in the incidence of retinal neovascular diseases such as diabetic retinopathy and age-related macular degeneration by 2020 [2]. Paradoxically, improvements in neonatal care have increased the survival of very premature infants who are at highest risk for a third neovascular disease, retinopathy of prematurity, contributing to increasing incidence in this condition that had previously been declining [3], [4]. This cumulative increase in vision loss due to various forms of retinal angiogenesis is becoming a significant public health problem [2]. While the initiating events in these diseases are unique, they each give rise to tissue hypoxia and high levels of angiogenic growth factors that trigger the pathologic overgrowth of new vascular networks that eventually obscure vision [5]. Treatments largely focuses on halting this process and include surgical resection of the neovasculature and laser photocoagulation of affected areas [6]. Recently, a regimen of frequent intravitreal injections of angiogenesis-inhibiting anti-VEGF reagents has proven to be a very successful treatment for age-related macular degeneration [7], [8] and shows promise for diabetic retinopathy [9]. However, complications associated with repeated injections and potential long-term secondary effects could potentially limit the utility of anti-VEGF therapy [10] and the fact that a percentage of patients do not respond [11] highlight the need to explore new target molecules for use in treatment of angiogenic-based diseases.

**Figure 1:**
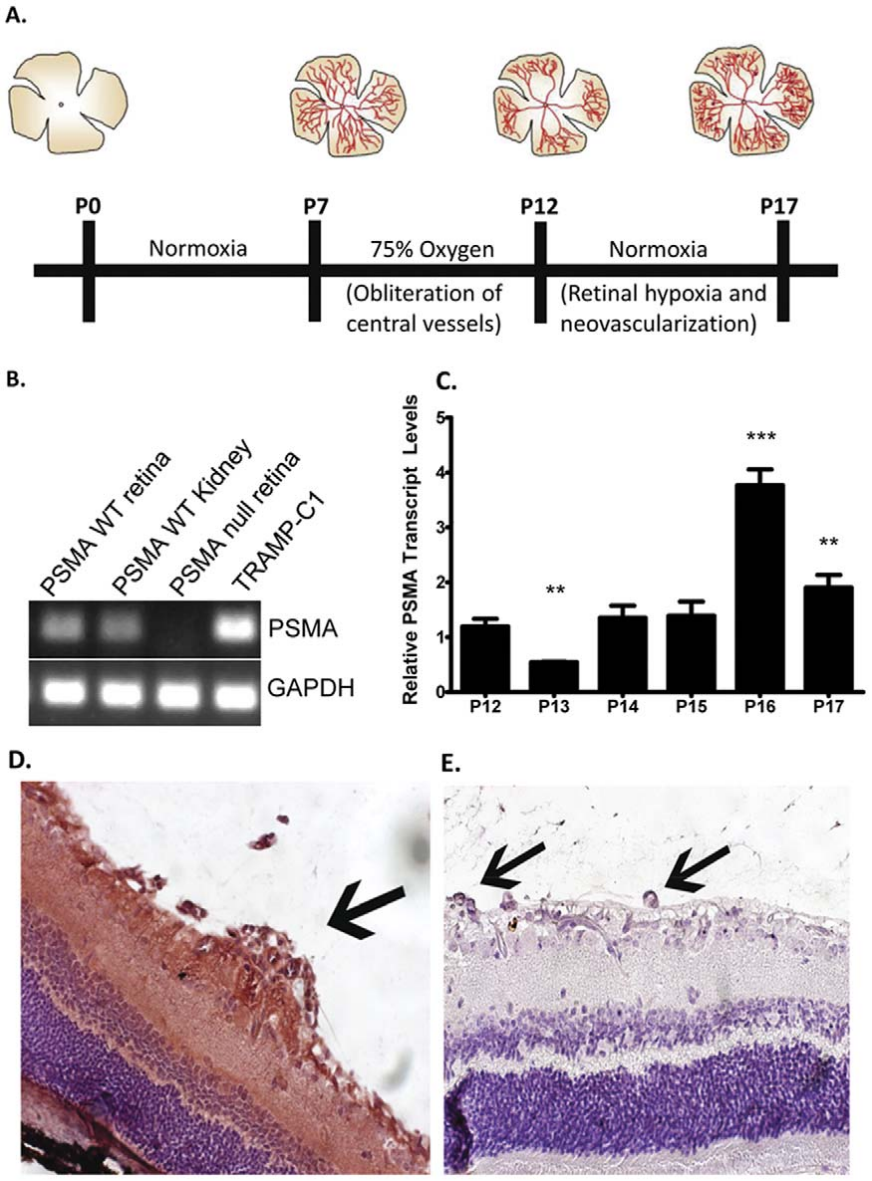
Retinal vasculature of wild type mice undergoing OIR expresses PSMA. **A**) A time line depicting the stages of retinal layer vascularization with diagrams of approximate vascular morphology of the retina at various time points. **B**) Conventional RT-PCR of RNA isolated from wild-type OIR retinas from P17 mice are positive for PSMA; PSMA wild-type kidney and TRAMP-C1 cells- PSMA positive controls, PSMA null retina- negative control. **C**) qRT-PCR for PSMA over time from retinal RNA isolated at the indicated time points relative to P12 levels. Paraffin embedded OIR retinas were immunostained for PSMA protein using the 3E2 antibody. Staining (red-brown) was observed on vascular tufts (arrows) of wild-type (**D**) but not PSMA-null (**E**) retinas. (n = 3 per group), **p,0.05, ***p,0.001.

The oxygen induced retinopathy (OIR) model evaluates angiogenic responses to tissue hypoxia and is an established animal model of retinal diseases resulting from dysregulated angiogenesis of the eye [5]. Mice are normally born with an immature retinal vasculature that progressively develops until approximately 3 weeks of age. Experimental exposure of neonatal mice to high levels of oxygen (75%) arrests the normal retinal vessel development and causes regression of existing retinal vessels. Upon subsequent exposure to normal oxygen levels (room air, 21%), tissues sense the relatively lower oxygen levels as a state of ‘relative hypoxia’. The retina is particularly sensitive to this change because of the initial loss of vessels during hyperoxia treatment and the normally well-regulated temporo-spatial signals that drive organized retinal vascular development are disrupted. In particular, the vessels in the periphery respond to this dysregulated hypoxic signaling by growing in a chaotic, disordered pattern while failing to revascularize the central retina, leaving an aberrant, avascular area (Figure 1A). The resulting retinal capillary network is tortuous, leaky and disorganized in the periphery while vessels of the central region are poorly perfused and largely nonfunctional, characteristic of the pathologic phenotype of retinal neovascular disease [12].

Prostate specific membrane antigen (PSMA) is a homodimeric type II transmembrane ectopeptidase with both folate hydrolase and N-acetylated, α-linked acidic dipeptidase or NAALADase activities [13]–[15]. In normal tissue, the expression of PSMA is predominantly restricted to prostatic epithelium, with low expression in kidney, salivary gland, duodenum and the central and peripheral nervous systems. As its name suggests, PSMA is highly over-expressed in prostate cancer where its increased expression correlates with advanced stages of prostate cancer and metastasis. Its function in the prostate remains unknown. Interestingly, PSMA has also been shown to be upregulated on the angiogenic vasculature of most solid tumors [16], [17]. Previous investigations in our lab have shown that mice lacking PSMA are incapable of mounting a pathologic angiogenic response *in vivo* suggesting that PSMA induction at angiogenic sites is critical for endothelial function in angiogenesis [18]. Mechanistically, we found that inhibition of PSMA significantly decreased Beta-1 integrin activation and reduced the subsequent downstream signal transduction cascades involving PAK and FAK, thus impairing the endothelial cell functions of adhesion, motility and invasion that are fundamental to the angiogenic response [18].

While angiogenesis is an important component in the progression of a number of diseases, it is clear that all angiogenic processes are not regulated by the same signals and are often distinct pathologies [19]. To determine if PSMA also contributes to angiogenesis in the retina, we studied the development of the retinal vasculature in wild type and PSMA null mice under physiological (normal retina development) and pathophysiological conditions (hypoxia-driven retinopathy).

## Results

In order to assess the role of PSMA in pathologic retinal angiogenesis, it was first necessary to verify that PSMA is expressed in the angiogenic neovasculature in wild type mice undergoing OIR. We initially evaluated retinal tissues from postnatal-day 17 mice (P17) when vasculoproliferative disease is maximal (Figures 1A and B). PSMA mRNA was detected in wild type P17 retinas as well as positive control kidney and the prostate tumor cell line TRAMP-C1 samples, but not in PSMA-null retinal tissue (Figure 1B). Subsequent qRT-PCR analysis showed that after an initial decrease, retinal PSMA mRNA expression is progressively induced over time, reaching maximum levels at P16 (Figure 1C). Immunohistochemical assessment confirmed that the angiogenic vasculature expressed PSMA protein where positive staining was detected on retinal angiogenic tufts (abnormal, glomerular-like, endothelial-rich capillary structures that extend beyond the inner limiting membrane into the vitreous of the eye, arrow, Figure 1D) in addition to the retinal neural tissue as previously reported [20]. No staining was observed on the extra-retinal vascular tufts in PSMA null OIR retinas (arrows, Figure 1E). Therefore, PSMA is expressed on the angiogenic neovasculature of wild-type mice undergoing OIR and may contribute to pathologic angiogenesis in the retina.

**Figure 2:**
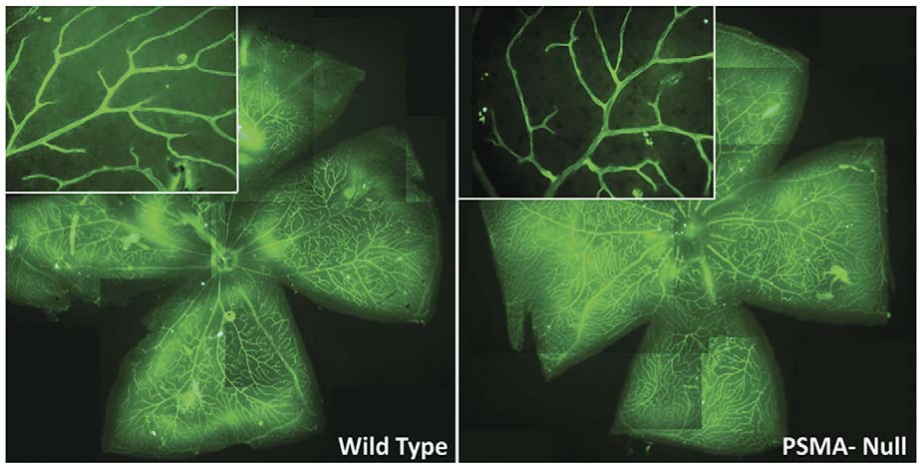
Retinal vasculature develops normally in PSMA null mice. Retinas from wild-type (**left**) and PSMA-null (**right**) mice (5X) raised in room air, harvested at P17 and perfused with FITC labeled *ricin communis* agglutinin 1 (RCA-1-FITC, green). Insets: higher (40X) magnification of the same retina and show normal radial branching pattern in animals of both genotypes. (Representative image, n = 3 per group).

Examination of the retinas of wild type and PSMA null mice demonstrated that the lack of PSMA does not affect normal developmental retinal angiogenesis (Figure 2). Wild type and null mice raised in normoxia were perfused with FITC-labeled *Ricin communis* agglutinin 1 (RCA-1-FITC) at postnatal day 17 to assess vessel integrity. Whole mounts of wild type and PSMA null retinas showed an indistinguishably normal pattern of radial vessels with patent, non-leaky, perfused vessels detected throughout the entire retinal area of both genotypes. Further magnification of the mid-peripheral region revealed a characteristically normal branched pattern of vessels in the ganglion cell layer of wild type and PSMA null retinas (Figure 2 insets). Based on these data, loss of PSMA does not affect the development of normal retinal vasculature, in agreement with a pathologic angiogenesis-specific role for this protein in vessel growth.

**Figure 3:**
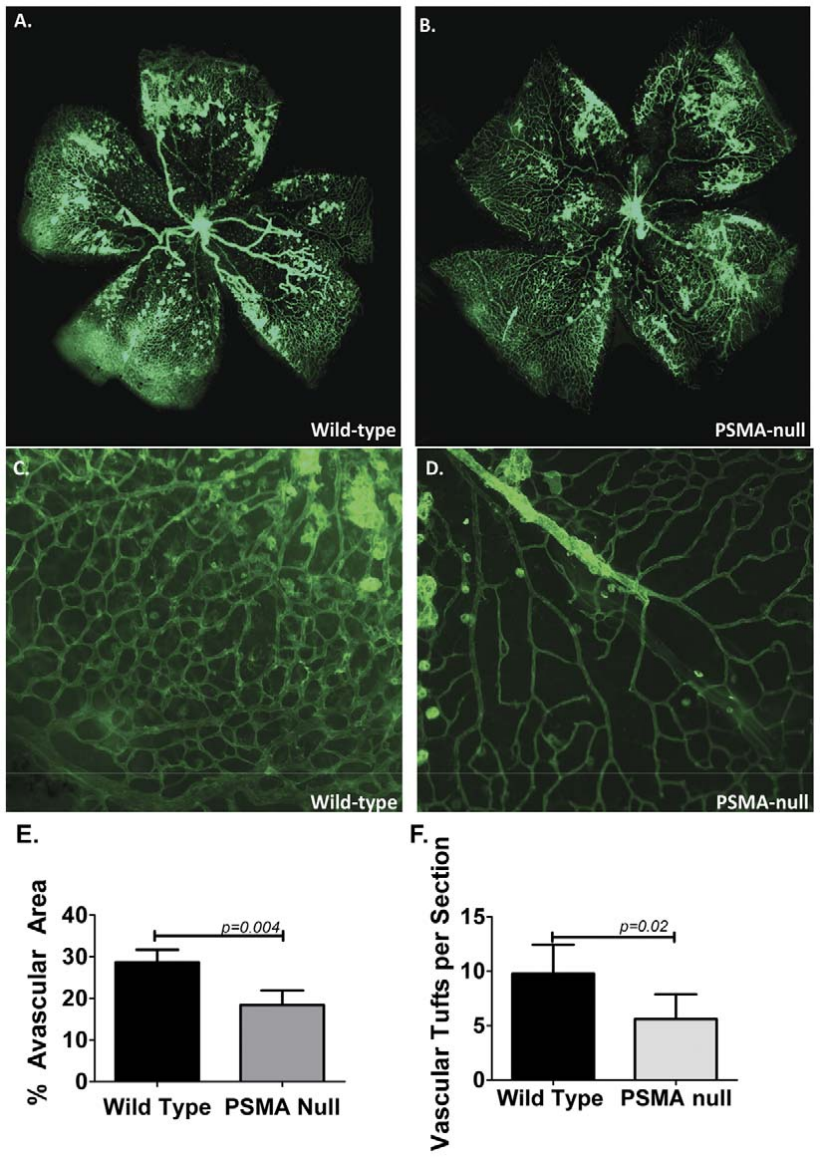
Pathologic angiogenesis is reduced in retinas of PSMA null mice. Whole-mount retinas from mice undergoing OIR were harvested at P17 and perfused with RCA-1-FITC. Wild type retinas (**A**) show a higher degree of central avascular area than retinas from PSMA null mice (**B**). Higher magnification (40x) of **BC** wild-type and **D**) PSMA-null capillary networks at the outer edge of the retina: Retinas isolated from wild-type mice **C**) show disorganized, tortuous vessels and vascular tufts while vessels of PSMA-null retinas **D**) are less tortuous and more closely resemble normal organization. **E**) Quantification of avascular area using Image J showed wild-type mice had an average avascular area of 28.7%, compared to 18.4% in the retinas isolated from PSMA null mice, (n = 4 per group, p = 0.004). **F**) Wild type animals had an average of 8.8 vascular tufts per histologic section, compared to an average of 5.2 tufts per section in PSMA-null (n = 8 per group, p = 0.017).

In contrast to the normal developmental vascular patterns found in PSMA null retinas, analysis of retinas harvested from mice undergoing OIR showed a striking phenotypic difference between wild type and PSMA null animals. Wild type and PSMA null mice were subjected to OIR and perfused with RCA-1-FITC lectin on postnatal day 17. As expected, wild type retinas displayed few vessels in the central area accompanied by an overgrowth of perfused vessels in the periphery (Figure 3A). Similarly, the capillaries in the mid-periphery were highly abundant and formed a dense, honeycombed pattern with closely spaced vessels (Figure 3C), rather than the radial, branched vascular pattern seen in untreated animals (Figure 2). In contrast, retinas isolated from PSMA null animals after OIR show a vascular pattern that more closely resembles the normal structure, with a relative reduction in avascular area in the central retina and a less dense, more highly branched capillary bed in the periphery (Figures 3B and 3D). Quantification of the relative degree of central retinal vascularity showed that PSMA null retinas had a significantly lower total avascular area than those from wild type animals (approximately 40%, Figure 3E). In addition to the decrease in retinal avascular area, the number of extra-retinal vascular tufts is significantly decreased in PSMA null animals compared to wild type controls (Figure 3F) suggesting a less pathologic angiogenic response in the absence of PSMA.

**Figure 4:**
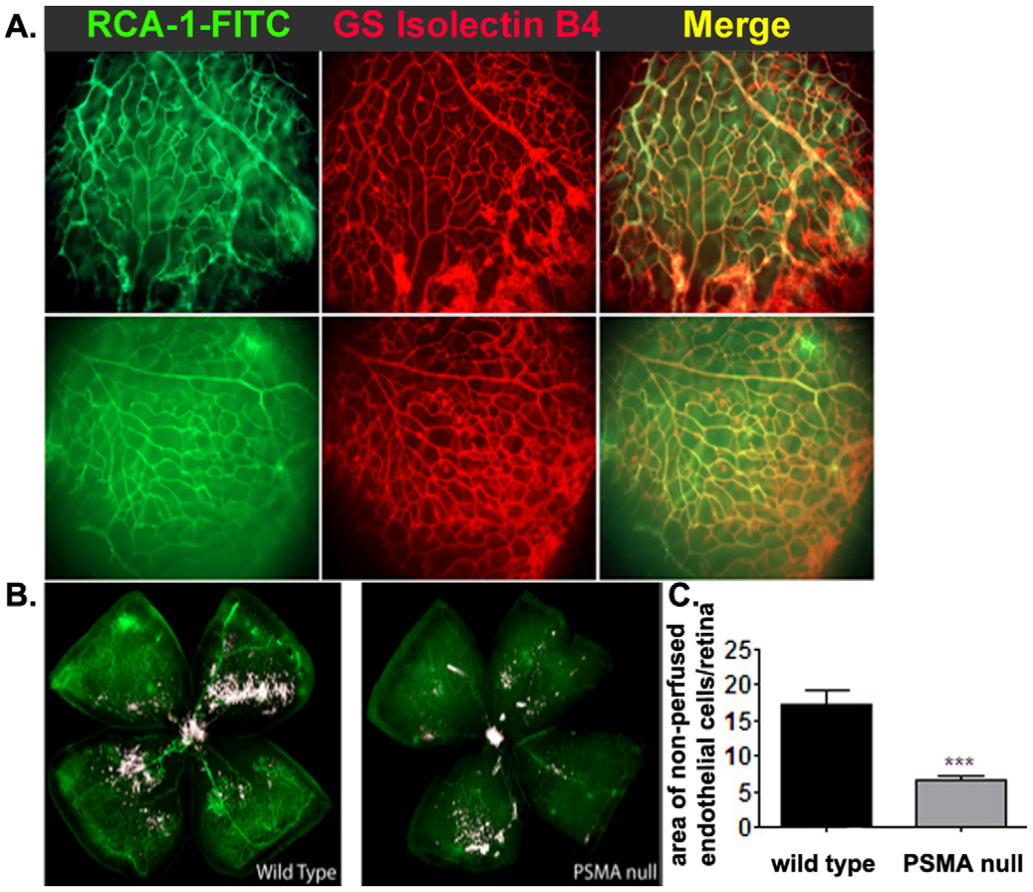
Perfusion of retinal vessels in PSMA null animals is increased. Following OIR, mice were perfused with RCA-1-FITC to detect perfused vessels immediately prior to sacrifice and harvest. Isolated retinas were then stained with Alexa 594 labeled *Griffonia simplicifolia* 1 isolectin B4 (GS Isolectin B4-A594) to stain all endothelial cells. **A**) RCA-1-FITC staining (**left, green**), GS Isolectin B4-A594 staining (**center, red**) and merged image (**right**) of representative wild type (**top**) and PSMA null (**bottom**) retinas. **B**) The double stained areas in the merged images (perfused vessels + endothelial cells) were pseudo-colored white to illustrate the area of non-perfused, GS Isolectin B4-A594 only staining endothelial cells (5X). **C**) Quantitative analysis of the relative extent of non-perfused endothelial cells per retina. (Representative image, n = 5 per group).

While vessels formed in response to OIR in PSMA null retinas appear more ‘normal’ and less pathologic with regard to microvascular patterning and vessel coverage than their wild type counterparts, it is important that these vessels function normally as well. To measure the integrity of the wild type and PSMA null vessels, we perfused mice undergoing OIR at P17 with RCA-1-FITC and then stained the harvested retinas with Alexa594 labeled- *Griffonia simplicifolia* 1 isolectin B4 (GSl-B4-A594 that specifically binds to endothelial cells [21]) to distinguish perfused, functional vessels (dual FITC and Alexa594 labeled) from non-perfused, nonfunctional endothelial clusters (single Alexa594 labeled). In both wild type and PSMA null animals, the RCA-1-FITC signals colocalized with GSI-B4 A594 staining to a certain degree, indicating that both wild type and PSMA null animals are capable of forming patent and perfused vessels (Figure 4A). However, quantification of the total area of non-perfused endothelial cells in retinas from both genotypes (RCA-1-FITC-negative/GSI-B4- Alexa594 positive, pseudo colored white in Figure 4B) illustrates that PSMA null retinas show half the amount of non-perfused endothelium as the wild type and the vessels formed are clearly more functional (Figure 4C). Thus, in addition to affecting both vascular density and patterning, vessels formed in the absence of PSMA appear to be better perfused, patent and function as normal vessels.

**Figure 5:**
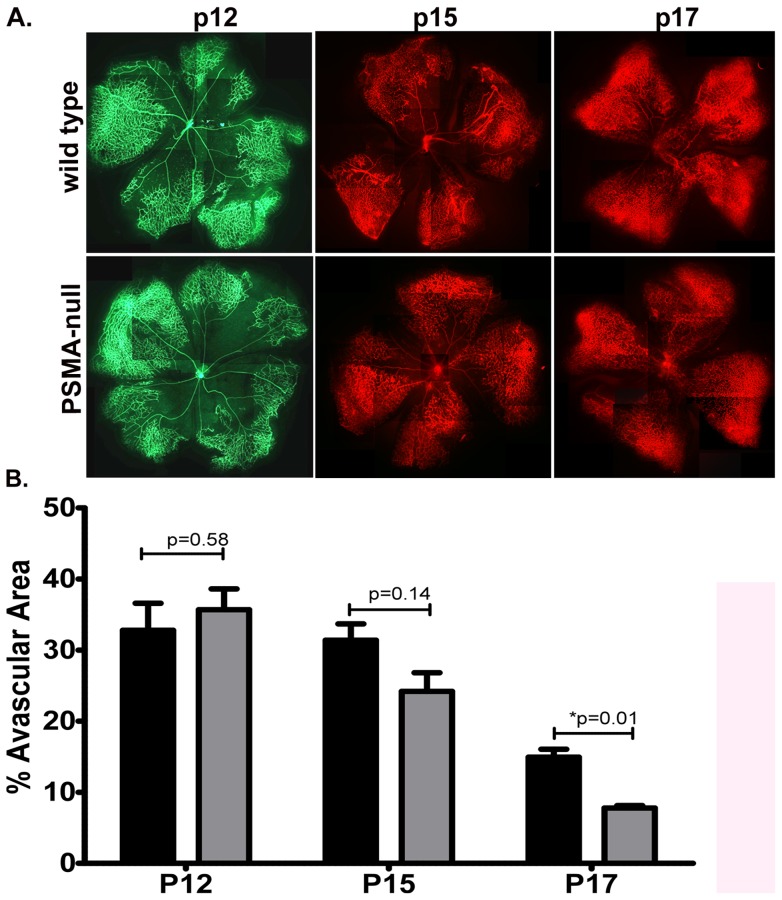
Vascular pathology is decreased in PSMA null animals undergoing OIR. **A**) Retinas were harvested from OIR mice at P12 (**left**), P15 (**center**) and P17 (**right**). Vasculature was stained using RCA-1-FITC (green, P12) or GS Isolectin B4-A594 (red, P15 and P17) and **B**) the central avascular area of each retina was measured using Image J. (n = 3 per group).

During OIR, vessels initially regress in response to the relatively hypoxic conditions experienced upon exposure to room air after high oxygen levels (P12). This regression phase is followed by a robust pathologic angiogenic response, producing the vessel overgrowth characteristic of retinopathy (P15–17). In principle, the phenotype in PSMA null OIR retinas could be due either to a defect in initial vessel regression or, more consistent with PSMA in angiogenesis, a limited pathological angiogenic response during the retinal revascularization phase. Retinas harvested from wild type and PSMA null mice undergoing OIR at the peak of vascular regression immediately after hyperoxia (P12), at the initiation of retinal revascularization (P15) or at the point of the most severe angiogenic pathology in the model (P17, Figure 5A) showed no statistical difference in avascular area on P12 or P15 (Figure 5B). However, measurements of the central avascular areas in retinas of PSMA null mice on P17 were significantly lower than wild type indicating that the attenuated retinal pathology in the PSMA null mice is not due to retinal vessel persistence during the vascular obliteration phase but rather to reduced pathological angiogenesis in response to relative hypoxia.

**Figure 6:**
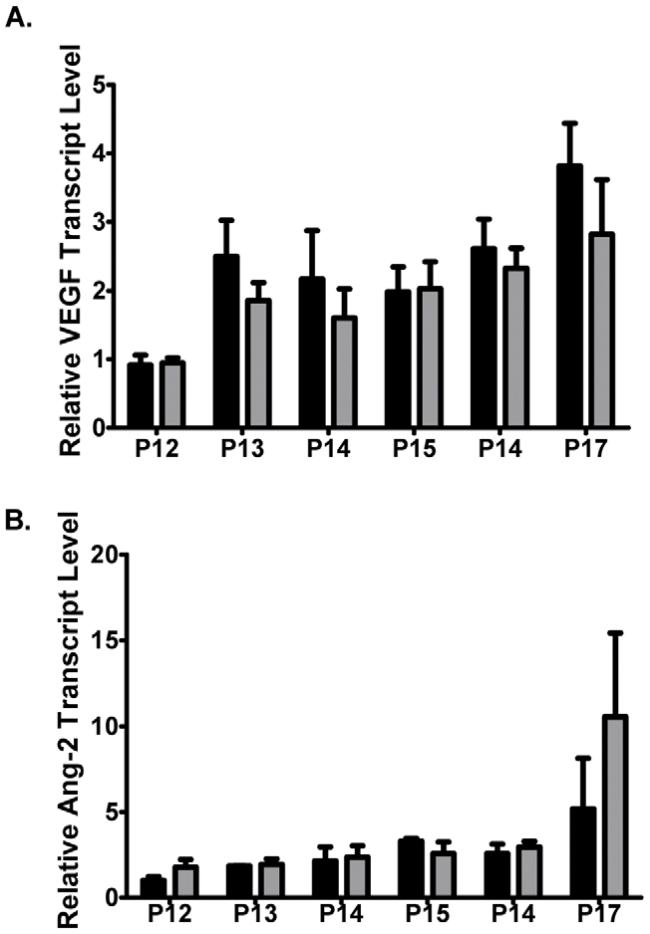
Angiogenic growth factor production is normal in response to hypoxia in PSMA null mice. PSMA expression was measured by qRT-PCR from RNA isolated from wild type and PSMA null retinas daily, beginning at the initiation of tissue hypoxia (P12 through P17). **A**) Wild type and PSMA mice produce similar VEGF expression levels during hypoxia treatment. **B**) PSMA null mice show similar Ang-2 levels to wild type mice over time. (n = 3 per time point).

Retinopathy is most commonly the result of uncontrolled VEGF expression that induces overgrowth of blood vessels that are generally immature, leaky, obstructed and torturous [6]. Changes in hypoxia and pH as a result of disease activate critical angiogenic signaling pathways culminating in the upregulation of VEGF [22], [23]. To establish if the decrease in angiogenic pathology observed in the OIR retinas of PSMA null mice was due to effects on hypoxic signaling, we examined the levels of VEGF and another hypoxia-induced angiogenesis promoting growth factor, angiopoietin 2 (Ang-2), over time using quantitative RT-PCR. In both wild type and PSMA null mice, VEGF levels increased in parallel by about 2.5-fold on P13 and remained elevated through P17 (Figure 6A). Similarly, lack of PSMA had no effect on Ang-2 levels which remained low until P16-17 and then increased by approximately 5–10 fold indicating that the blunted pathologic angiogenesis in the PSMA null mice is not due to deficiencies in production of pro-angiogenic factors in response to hypoxia (Figure 6B) and, consistent with our previous published data [18], is the result of an endothelial cell-intrinsic defect.

**Figure 7:**
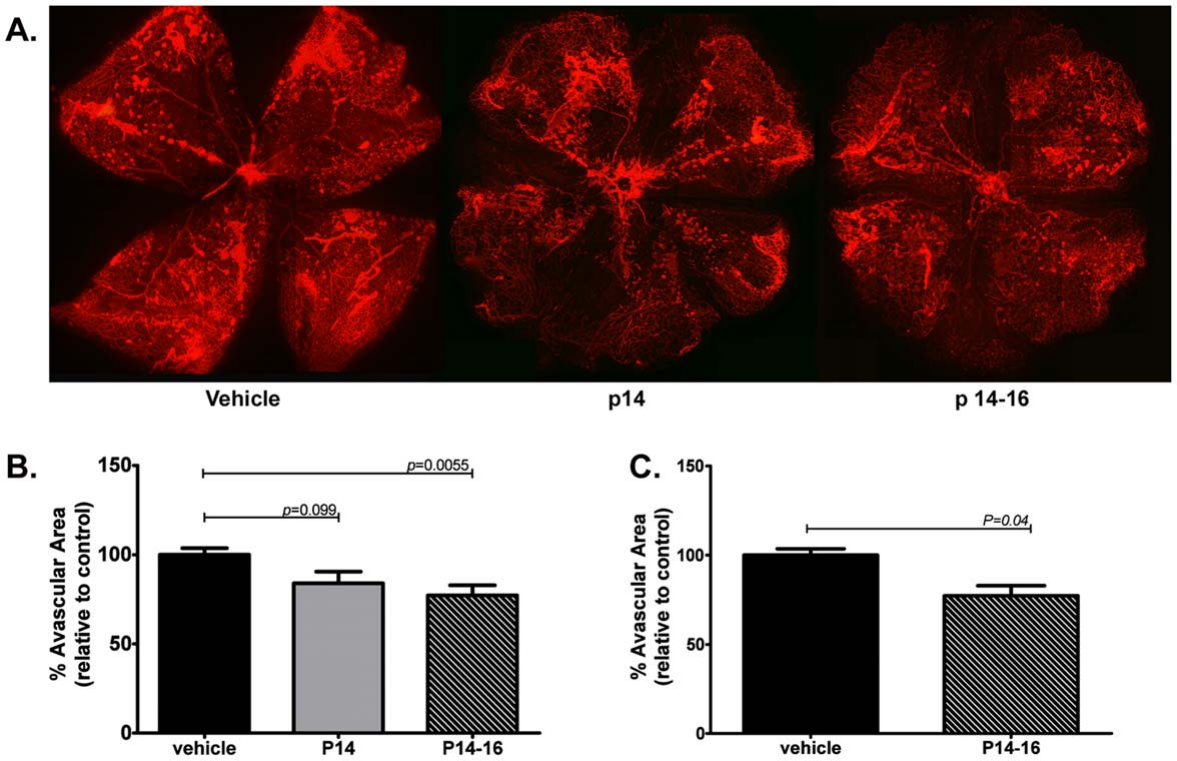
Inhibition of PSMA decreases pathologic angiogenesis in a mouse model of OIR. **A**) Wild type mice undergoing OIR were treated systemically with either a single dose of 100mg/kg 2-PMPA on P14 (n = 3, **center**) or three daily doses from P14 through P16 (n = 6, **right**), controls received vehicle (PBS, **left**). Retinas were isolated on P17, vasculature was stained using GS Isolectin B4–A594 and the avascular area calculated using Image J software. **B**) Mice receiving a single dose on P14 showed a slight but not statistically significant decrease in avascular area, whereas mice treated from P14–16 showed a significant decrease (23%) in avascular area compared to vehicle treated controls (p = 0.0055). **C**) Wild type mice on the OIR protocol were treated once on P14 with 10mg intravitreal 2-PMPA (10mg/ml, 1mL) in one eye and 1mL Vehicle (PBS) in the contralateral eye. Retinas treated with intravitreal 2-PMPA showed a significant decrease (16.66%) in avascular area compared to control retinas.

Finally, to determine the therapeutic potential of PSMA as a target in retinopathies, we treated wild type mice undergoing OIR with the small molecule PSMA inhibitor 2-PMPA in two dosing protocols, systemic administration of 100mg/kg 2-PMPA either once on P14 or daily from P14-P16 (Figures 7 A, B). Wild-type mice treated with a single dose of inhibitor showed a slight but not significant decrease in avascular area compared to vehicle control. However, daily administration of the inhibitor over three days decreased revascularization, as measured by a significant increase in avascular area compared to control and suggests that PSMA inhibition may be a viable therapy for slowing the progression of retinopathy. However, since PSMA is expressed in other tissues such as the brain where it regulates glutamate levels, systemic PSMA inhibition may potentially produce harmful side effects. To avoid this possibility we treated OIR mice with a single intravitreal injection of 2-PMPA on P14 and measured avascular area three days later. Indeed, retinas of inhibitor-treated mice showed improved vascular coverage and reduced avascular area than the retina from the vehicle control (Figure 7C). Although intravitreal injection is not as effective as systemic treatment in our experiments, it is likely that multiple doses would increase its effectiveness. While repeated injections in the mouse eye caused significant scarring, this regimen is well tolerated by humans and is the current method of anti-VEGF treatment and could be adapted for administration of PSMA inhibitors.

## Discussion

Histological observations of high PSMA expression on the angiogenic neovasculature of solid tumors suggested that it may regulate endothelial function [24], [25] and prompted our earlier investigation into the function of PSMA in angiogenesis [18]. In the previous study we demonstrated that tumor angiogenesis was significantly impaired in PSMA null mice due to diminished Beta-1 integrin activation and a subsequent decrease in endothelial cell invasion, thus affecting angiogenesis. In the current investigation we extended these observations to examine if PSMA may be a viable target for treatment of dysregulated angiogenesis that is the underlying cause of the majority of devastating retinopathies and may be of benefit to the large proportion of patients that are refractory to current anti-VEGF regimens [8]. To evaluate the role of PSMA in retinal neovascularization, we used a reliable model of retinal angiogenesis (oxygen induced retinopathy or OIR) that mimics many aspects of proliferative retinopathies [5]. We initially determined that PSMA expression is induced in the retinal vasculature undergoing OIR, indicating that it may indeed contribute to pathology in this model. We also observed that while the retinal vasculature develops normally in mice lacking PSMA, upon OIR challenge the lack of PSMA results in significant reduction of pathologic vascular angiogenesis when compared to wild type animals. The newly formed vessels in the PSMA null retinas were highly organized with regular microvascular patterning in contrast to the disorganized, chaotic and tortuous, characteristically angiogenic, vessels present in wild type retinas. Furthermore, lack of PSMA resulted in a reduction of avascular area and number of vascular tufts, indicating a diminished pathological response. Perfusion with fluorescent lectin showed that the retinal vessels in PSMA null animals were significantly more patent and well perfused than those of wild type mice, reinforcing the notion that PSMA regulates angiogenic vessel formation and its loss promotes more ‘normal’ vascular growth. Similarly, this response was not due to effects on angiogenic growth factor production or effects on vessel regression in response to high oxygen levels. Finally, systemic or intra-ocular administration of an inhibitor of PSMA enzymatic activity phenocopied the response of null animals and resulted in a marked decrease in avascular area and increased vessel coverage in treated animals. Taken together, these results support a role for PSMA in retinal angiogenesis and thus may be a valuable therapeutic target.

The mouse model of OIR used in this study has been well characterized and has both strengths and limitations. The model lends itself well to genetic manipulation, either by deletion or overexpression, to test the contribution of specific genes on retinal neovascularization [26], [27]. In addition, these models are quite reproducible, cost-effective and quantitative analysis is reliable with relatively low variability [28]. However, in human retinopathy of prematurity, the peripheral vessels are destroyed by exposure to high oxygen levels while the central vessels are affected in the mouse model [27]. However, despite this discrepancy the mouse OIR model recapitulates the pathology of progression resulting from ischemia-induced neovascularization to a remarkable degree, and thus is a reliable and robust model of this condition [26].

While numerous molecules and signaling pathways are responsible for developmental angiogenesis (reviewed in ref [29]), perhaps the best characterized system is that of VEGF, which through alternative splicing and proteolytic processing mechanisms, produces various VEGF isoforms that differ in their ability to bind heparan sulfate residues on extracellular matrix and cell surface proteins. Proper positioning of these isoforms creates precise gradients of matrix-bound and freely diffusible angiogenic factors that guide and control the endothelium of the developing vasculature. Perfusion of the vessels and pericyte coverage contributes to cessation of angiogenesis, leading to normal, quiescent vasculature [30]. In contrast, while many of the same factors that regulate developmental angiogenesis are responsible for the blood vessel formation in pathologic states, under the pro-angiogenic conditions of sustained hypoxia, infiltrating immune cells and tissue damage the tight regulation of vessel growth is lost resulting in an asynchronous, leaky and immature vasculature and persistent angiogenesis. Clearly, additional unique mechanisms must regulate pathological angiogenesis since similar to PSMA, a number of pro-angiogenic molecules (such as PlGF [31], PECAM-1 [32], aminopeptidase A [33], CD13 [34]) have been shown to be dispensable for developmental angiogenesis. This suggests that they participate solely in the pathological response, making them particularly promising therapeutic targets.

Consistent with our previously published data linking PSMA regulation of angiogenesis to endothelial Beta-1 integrin signaling and adhesion [18], the decrease in pathologic angiogenesis in the retinas of PSMA null mice was not due to deficient VEGF or Ang-2 production in response to hypoxia. In fact, the retinas of PSMA null animals undergoing OIR displayed levels of VEGF and Ang-2 similar to those of the wild type, but angiogenesis was decreased in the absence of PSMA indicating that PSMA participates in endothelial cell functions downstream of angiogenic growth factor signaling.

While it is clear that VEGF-induced signals are critical for angiogenesis, proper interactions of endothelial cells with extracellular matrix proteins via the integrins are equally as important to promote migration and other cellular processes essential to the angiogenic response [35]–[37]. Numerous studies have implicated various integrin pairs in retinal vascular pathology [19], [38]–[41], where blocking, disruption or lack of integrins generally impairs angiogenesis, supporting PSMA regulation of integrin signaling as the mechanistic basis for our findings. Importantly, the signals initiated by integrin/extracellular matrix and growth factor/receptor interactions are coordinated and transmitted to intracellular cytoskeletal and signaling proteins by the focal adhesion kinase FAK. Thus FAK controls essential cellular processes such as growth, survival, migration and differentiation [42]. Pertinent to this study, FAK has been previously demonstrated to be involved in pathological retinal angiogenesis [43]. In this study, overexpression of FAK by intra-ocular injection of an expression plasmid into mice undergoing OIR led to the formation of large retinal vascular tufts and pathologic neovascularization, whereas injection of a plasmid expressing the FAK inhibitor FRNK decreased new vessel formation [43]. Combined with our previous data [18], the current study suggests that PSMA likely regulates both retinal and tumor angiogenesis via the same or similar mechanisms, by enhancing adhesion and activation of Beta-1 integrin and increasing its associated FAK signaling, thus contributing to endothelial cell responses to angiogenic signals in a manner independent of VEGF signal transduction.

Due to its widespread contribution to many pathological disorders, early predictions were that angiogenesis would be a particularly effective therapeutic target. However, the results of clinical trials evaluating the efficacy of modulators of angiogenesis, primarily by blocking the VEGF pathway, in the treatment of cancer, diabetic retinopathy, rheumatoid arthritis, age-related macular degeneration and cardiovascular disease suggest that more precisely targeted therapies or therapies directed at multiple angiogenic pathways are needed to improve the treatment of angiogenesis-associated diseases [44]. Similarly, while recurrent intravitreal injections of the monoclonal anti-VEGF antibody ranibizumab (Lucentis) is the standard of care for age-related macular degeneration where the majority of patients show visual stabilization [8], isolated cases of macular ischemia and persistent elevation in intraocular pressure following treatment have been reported [45], [46] and the long term effects of drug resistance, tolerance or ancillary effects in targeting this pathway have yet to be addressed. Furthermore, additional treatment modalities are clearly needed for those patients who do not respond to this therapy. In this regard, PSMA is also involved in the pathogenesis of neurodegenerative diseases by releasing free glutamate from N-acetyl-aspartyl-glutamate (NAAG) leading to neurotoxic levels of glutamate or ‘glutamate excitotoxicity’ [47], [48]. Small molecule inhibitors of PSMA, including 2-PMPA, provide protection from glutamate excitotoxicity in animal models of stroke, amyotrophic lateral sclerosis, neuropathic pain and diabetic neuropathy [49], [50]. While supportive of PSMA as a potential therapeutic target, 2-PMPA is a highly polar molecule, which limits its utility as a therapy. However, other more feasible PSMA inhibitors have been produced and one has been shown to be safe and well tolerated by humans at doses shown to be effective in animals [51]. These promising drugs are currently awaiting clinical trials [50], [52]. Our finding that 2-PMPA decreased retinal vascular pathology in wild type mice undergoing OIR strongly suggests that PSMA inhibition may be a novel treatment that could be rapidly translated into therapeutic use for retinal diseases. In addition, due to poor drug permeability across the blood retinal barrier, intraocular injection is the preferred method of administration for age-related macular degeneration and so confounding variables such as long-term effects of PSMA inhibition on glutamate levels in the brain and bioavailability would be minimized. Therefore, our data indicate that inhibition of PSMA offers a VEGF-independent means of regulating retinal vascularization and perhaps in combination with other therapies may be a new and attractive target to improve the treatment of retinal neovascular diseases such as age-related macular degeneration.

## Methods

### Ethics Statement

The University of Connecticut Health Center has been fully accredited by the Association for the Assessment and Accreditation of Laboratory Animal Care International (AAALAC) since June 21, 1977. Most recent full accreditation: July 8, 2010. Animal Welfare Assurance number A3471–01; Valid Through: April 30, 2014. All animals were used according to specific animal protocols approved by the UCHC Institutional Animal Care Committee, UCHC.

### Mice

C57/BL6 PSMA null mice were produced in 2005 (11) and have been backcrossed extensively to C57Bl/6. These were a generous donation from Warren Heston at Cleveland Clinic. Sibling heterozygotes were bred to produce the parents of (wild type × wild type) and (null × null) mating units and pups were no more than 3 generations removed from these units. Littermate progeny of the (het × het) crosses were used to breed the test animals in the UCHC animal facility and were provided food and water *ad libitum*. Littermates were compared in all experiments.

### Oxygen Induced Retinopathy (OIR) Model

Seven day old mice weighing 6g or greater and their lactating mothers were maintained in 75% oxygen for five days. After 2.5 days in 75% oxygen, the lactating females were replaced with surrogate dames. Mice were then returned to room air (relative hypoxia) for up to 5 days. When indicated, mice were anesthetized with Tribromoethanol and perfused with FITC labeled *Ricin communis* agglutinin 1 (RCA-1-FITC) that binds preferentially to galactose containing oligosaccharides, to label perfused capillaries before enucleation. Eyes used for retinal whole mounts were pre-fixed in 4% paraformaldehyde (PFA) on ice 30 minutes, retinas isolated, then post-fixed 45 minutes in 4% PFA. After rinsing with PBS, retinas were blocked with 10% normal goat serum in PBS for 1 hour and stained overnight 4°C with the required marker or antibody. Retinas used for RNA isolation were stored in RNAlater on ice and the RNA was isolated using Qiagen RNA isolation kit according to manufacturer's instructions.

### Quantitative analysis of retinal vascularization

Eyes used for histological sectioning were fixed overnight in 4% PFA at 4°C, stored in 70% ethanol, paraffin embedded, and 6µM sections cut. To analyze the number of extra-retinal vascular tufts, eyes were sagitally embedded in paraffin and sectioned 6µM apart. Each eye was analyzed using 5 H&E stained sections on either side of the optic nerve; vascular tufts, defined as endothelial cells on the vitreal side of the inner limiting membrane, were quantified under 40x by a blinded observer. To quantify avascular area, retinal whole mounts were blocked using 5% normal goat serum (Zymed), then stained overnight at 4°C with 1microgram/mL of endothelial cell specific Alexa594 conjugated *Griffonia simplicifolia* 1 isolectin B4 (Invitrogen I21413). After mounting whole mounts on slides, retinas were imaged using a 5× objective on a Zeiss LSM Confocal microscope and an image of the entire retina obtained using the tile-scan feature. Central avascular area was outlined in NIH Image J using polygonal select tool by a blinded observer. Avascular area and total retina area were calculated using area measure tool and avascular area/total retinal area was calculated. Branch points were quantified in a 40× image of the outer edge of a retinal leaflet.

### Immunohistochemistry

Slides were deparafinized and rehydrated. Antigen retrieval was conducted using 10mM sodium citrate (pH 6.0) in a pressure cooker. Endogenous peroxidase activity was quenched by incubating slides 15′ in 0.3% H_2_O_2_. Slides were blocked in 1% BSA for 30 minutes at room temperature in a humidified chamber then incubated under 1∶50 3E2 antibody overnight in a humidified chamber. Slides were washed 3 times in PBS. 1∶500 biotinylated secondary antibody (Vector Labs goat anti-mouse BA9200) in 1% BSA was applied and slides incubated for 1 hour at room temperature in a humidified chamber. Slides were washed 3 times, Vectastain Elite ABC Kit (Vector Labs SK-6100) was applied for 30 minutes. Slides were washed once in buffer, once in H_2_O, then Novared (Sigma D-4293) was applied for 5 minutes. Slides were washed in H_2_O, counterstained with hematoxylin (Vector Labs H-3404), washed in H_2_O, dehydrated and mounted under Cytoseal 60.

### Quantitative RT-PCR

RNA was isolated from retinas using Qiagen RNA isolation kit according to manufacturer's instructions. cDNA was generated using BioRad iScript reagent, including a reaction with no reverse transcriptase to control for DNA contamination. qPCR reactions were performed in triplicate using BioRad iQSupermix as indicated in manufacturer's directions and an Eppendorf thermocycler. Primers used in qPCR are as follows: **cyclophilin A** forward: 5′- ATGGCAAATGCTGGACCAAA-3′; reverse: 5′- TGCCATCCAGCCATTCAGT-3′; **VEGF** forward, 5′- CACGACAGAAGGAGAGCAGAAGT-3′, reverse, 5′- TTCGCTGGTAGACATCCATGAA -3′; **PSMA** forward: 5′- GATGTAGTGCCACCATACAGTG-3′, reverse: 5′- GCCAGTTGAGCATTTTTAACCAT 3′; **Ang-2** forward, 5′- TCAACAGCTTGCTGACCATGAT-3′, reverse, 5′- GGTTTGCTCTTCTTTACGGATAGC-3′. All experimental gene levels were normalized to a cyclophilin A internal loading control. Fold change calculations were performed using the wild-type P12 retina sample as control.

### PSMA Inhibition Studies

On P15 mice were anesthetized using 12.5 mg/mL Avertin (250 mg/Kg). Using a dissecting microscope, the eyelids are opened using a sterile scalpel or by gently teasing eyelid apart using jewelers forceps. The tip of a 33 gauge needle attached to a Hamilton syringe was positioned adjacent to the pars plana, 2.5 mm posterior to the limbus, and 1uL or 10ug/uL or 1ug/uL 2-PMPA dissolved in PBS was injected into the vitreous cavity. The needle was kept in place for at least 20 seconds before being removed to prevent leakage. The eyelids were approximated over the eye and antibiotic ointment was applied. The contralateral eye was injected with sterile PBS as a control. Mice were monitored until regaining consciousness then returned to the home cage. For systemic inhibition studies, 100mg/kg or 50 mg/kg 2-PMPA (Alexis) in sterile PBS or PBS alone was injected intraperitoneally on days indicated.

### Statistics

Statistical differences were assessed using the 2-tailed Student's t test. *P* values less than 0.05 were considered significant. All error bars represent standard deviation.
