# Correction: Prostate Specific Membrane Antigen (PSMA) Regulates Angiogenesis Independently of VEGF during Ocular Neovascularization

**DOI:** 10.1371/annotation/a66c73f8-4cef-454c-9595-11f657ade2bc

**Published:** 2012-11-27

**Authors:** Christina L. Grant, Leslie A. Caromile, Khayyam Durrani, M. Mamunur Rahman, Kevin P. Claffey, Guo-Hua Fong, Linda H. Shapiro

Vivienne Ho was not included in the author byline. She should be listed as the third author and affiliated with Center for Vascular Biology, University of Connecticut Health Center, Farmington, Connecticut, United States of America. The contributions of this author are as follows: Conceived and designed the experiments, performed the experiments, analyzed the data, and contributed reagents/materials/analysis tools. 

